# Sad State of Phage Electron Microscopy. Please Shoot the Messenger

**DOI:** 10.3390/microorganisms2010001

**Published:** 2013-12-24

**Authors:** Hans-W. Ackermann

**Affiliations:** Department of Microbiology-Immunology and Infectiology, Medical School, Laval University, Quebec, QC G1K 7P4, Canada; E-Mail: ackermann@mcb.ulaval.ca; Tel.: +1-418-656-2131 (ext. 2558); Fax: +1-418-656-7698

**Keywords:** bacteriophage, contrast, electron microscopy, positive staining, quality control

## Abstract

Two hundred and sixty publications from 2007 to 2012 were classified according to the quality of electron micrographs; namely as good (71); mediocre (21); or poor (168). Publications were from 37 countries; appeared in 77 journals; and included micrographs produced with about 60 models of electron microscopes. The quality of the micrographs was not linked to any country; journal; or electron microscope. Main problems were poor contrast; positive staining; low magnification; and small image size. Unsharp images were frequent. Many phage descriptions were silent on virus purification; magnification control; even the type of electron microscope and stain used. The deterioration in phage electron microscopy can be attributed to the absence of working instructions and electron microscopy courses; incompetent authors and reviewers; and lenient journals. All these factors are able to cause a gradual lowering of standards.

## 1. Introduction

The recent impressive advances in cryo-electron microscopy (cryoEM) and three-dimensional image reconstruction suggest that all is well in phage electron microscopy. The reality is very different. CryoEM, although producing spectacular images and a wealth of information, is not a routine technique, but is limited to the study of selected viruses in specialized laboratories and generates not so much virus micrographs as colored drawings. The real issues are common transmission electron microscopy (TEM) and negative staining of isolated viruses on continuous carbon films, which generate about 100 phage descriptions per year [1].

Negative staining by phosphotungstic acid (PT) and other substances, such as AgNO_3_, was introduced in 1955 into virology [2]. PT staining was first applied in 1959 to bacteriophages [3,4], soon to be followed by uranyl acetate (UA) in 1961 [5]. From the outset, negative staining produced produced spectacular images. It is arguably the fastest technique in virology, is indispensable for virus description and classification, and provides instant diagnosis of virus families and often genera and species. The very first images of phage T2 in 1959 [4] were of high quality and indeed superior to many phage pictures published today. Virus electron microscopy (EM) peaked in 1968-1982 with the publication of classic atlases of animal, bacterial, and plant viruses [6,7,8] and a book on phage ultrastructure [9]. Today, thousands of viruses have been examined in the electron microscope, including at least 6300 viruses of bacteria and archaea [10].

After 1985, “manual” electron microscopes were increasingly replaced by “digital” instruments. This meant that darkrooms, films and photographic paper gradually disappeared and were replaced by digital cameras and electronic image acquisition and software editing. About 15 years ago, I noticed a decline in bacteriophage electron microscopy [10]. This decline now resembles a free fall. I was introduced to electron microscopy (EM) in 1957–1958 as a student, started research into phage EM in 1967, and examined some 1800 phages, thus probably more than any living person to date. As a chairman of the Bacterial Virus Subcommittee of the ICTV (International Committee on Taxonomy of Viruses) for some 15 years, preparing periodical counts of prokaryote viruses [1] and setting up a comprehensive phage bibliography of now 33,000 references [11], I had many reasons to follow the development of phage electron microscopy. In order to discuss the present state of phage electron microscopy, it is important first to assess the quality of images and see whether it correlates with image origin and the types of electron microscopes used.

## 2. Publications and Criteria for Analysis

This survey covers 256 articles and four books with micrographs of prokaryote viruses, published between 2006 and 2012. It is believed to be complete. The material was analyzed with respect to geographical origin, journals, and electron microscopes. Criteria used were (1) contrast, sharpness, size of particles and images, and (2) information on magnification control, types of electron microscopes, and stains.

### 2.1. General

Publications were subjectively rated as poor (168 or 65%), mediocre (21 or 8%), or good (71 or 27%). Although most poor micrographs or articles were easily identified, categories were not always clear-cut; for example, a good micrograph could be accompanied by a poor description of methods or viruses. Also, good and poor images were sometimes present in the same article. It was not attempted to determine the exact reasons for unsharp images because they are (1) countless and (2) rarely evident in the published micrographs, mainly due to the small sizes and low magnification of many pictures. Only strong astigmatism could be diagnosed with some confidence. Poor images generally do not show details such as tail striations, tail fibers, and collars. Their frequency seems to be on the increase.

### 2.2. Countries of Origin

The articles or books originated from 37 countries, P.R. China and Taiwan being considered as different entities. Most were from the USA (37), South Korea (29), and P.R. China (28). Many countries contributed a single publication with phage images. Good and poor phage images were produced almost everywhere; however, those from East Asia, especially South Korea, were often unsatisfactory and sometimes miserable. The number of publications frequently reflected the activity of a single laboratory. See details in Table 1.

**Table 1:** Origin of publications.

Country	Articles			
	Overall	Poor	Mediocre	Good
Australia	8	8	-	-
Canada	22	9	1	12
China (P.R.)	28	24	3	1
Finland	12	3	-	9
India	12	6	2	4
Japan	9	6	2	1
Poland	6	4	-	2
Russia	12	5	3	4
South Korea	29	28	-	1
Switzerland	7	3	1	3
United Kingdom	17	11	1	5
USA	37	26	2	9

More articles, mostly of poor quality, were published in: Argentina, Belgium, Brazil, Chile, Czechia, France, Germany, Ireland, Israel, Italy, Lithuania, Mexico, New Zealand, Nigeria, Norway, Pakistan, the Philippines, Portugal, Serbia, South Africa, Spain, Sweden, Taiwan, Thailand and the Ukraine.

### 2.3. Journals

The 256 journal articles appeared in no less than 77 different periodicals from all over the world. Most were from the USA, Germany, and the United Kingdom. *Applied and Environmental* *Microbiology* and *Archives of Virology* were by far the leading journals in publishing phage micrographs. Overall, ecologically oriented journals predominated because they tended to accept purely descriptive manuscripts. Good and poor electron microscopy was found everywhere, suggesting that no journal exercised any particular quality control. See details in Table 2.

**Table 2:** Principal journals featuring phage micrographs.

Articles	Journal
33	*Applied and Environmental Microbiology*
27	*Archives of Virology*
17	*Journal of Virology*
12	*Journal of Bacteriology*
10	*Environmental Microbiology*
8	*Current Microbiology*, *Virological Journal*
7	*Microbial Ecology*
6	*Virology*
5	*Bacteriophage*, *Microbiology-UK*

### 2.4. Electron Microscopes

A bewildering variety of about 60 models or types of electron microscopes was used. They were from four major manufacturers [Hitachi (Tokyo, Japan), 11 models; JEOL (Tokyo, Japan), 22; Philips/FEI (Hillsboro, OR, USA), 19; Zeiss (Oberkochen, Germany), 9]. Models from minor manufacturers, e.g., Tesla (Tescan, Brno, Czech Republic), had disappeared from publications during the survey. The number of some 60 models is a minimum as some instruments were incompletely identified (e.g., simply as “Morgagni, Tecnai, G2 Twin”). Sometimes, especially in low-grade publications, they were not even named. The vast majority were “digital” electron micoscopes. The author is partial to Philips/FEI instruments, but insists that any type of electron microscopes can give good or poor images. However, he notes that poor images are uncomfortably often associated with the JEOL 1200EX. The great variety of electron microscopes in use or on the market precludes the creation of universal EM courses and instruction manuals. See details in Table 3.

**Table 3:** Electron microscopes used.

Manufacturer	Model	Number	Model	Number	Model	Number
Hitachi	H-300	1	H-7100	7	H8100S	1
	H-300TM	1	H-7500	5	H8100	1
	H-600	1	H-7600	1	800 STEM	2
	H-7000	1	H7650	1	-	-
JEOL	7A	1	1010	3	1230	4
	100C	1	1011	2	1400	5
	100CX	3	1200	1	2000	3
	100S	2	1200EX	7	2000EX	1
	100SX	1	1200EXII	14	2010	1
	100SXII	2	1210	1	2010HC	1
	200CX	1	1220	1	2011	1
	210	1	-	-	-	-
Philips/FEI	100	1	400T	1	T10	3
	201	1	400HGM	2	T20	1
	201C	1	Morgagni 268	1	T30	1
	205	1	CM12	3	G2 Spirit TWIN	8
	208S	1	CM100	6		
	300	19	CM120	2	G2 Spirit Bio TWIN	10
	400ST	1	F20	1		
Zeiss	EM10	2	Leo 902	14	Leo 912AB	3
	EM109	1	Leo 910	1	Libra 120	1
	Leo 900	1	Leo 912	2	Supra 40VP	1

## 3. The Problems

### 3.1. Technical Problems

(1) The main problem is poor contrast. It is pervasive and international, found in “manual” and “digital” electron microscopy and in micrographs from any countries and journal studied. It is probably the single major reason why many present-day electron micrographs appear as objectionable. Very simply, images are often grey on grey. This is unacceptable and such images should be automatically rejected. Both uranyl acetate (UA) and phosphotungstate (PT) can give highly contrasted micrographs. In my experience, ammonium molybdate, though an excellent stain, gives good but rather low-contrast images.

“Manual” electron microscopy, by means of selection of papers, developers, and, above all, optical filters allows for considerable image improvement in the darkroom. This is impossible with “digital” electron microscopes where contrast is created in the moment of image acquisition by adjusting, by trial and error, the histogram that comes with the camera software [12]. Unfortunately, the central sections of this histogram give flat pictures with little contrast. Regrettably, company prospects and manuals are, to my knowledge, totally silent on the subject of contrast and do not provide any guidance. As evidenced in these prospects and an inordinate amount of contrastless images in the recent literature, poor contrast seems to be a general problem of “digital” EM, but is generally glossed over. For unclear reasons, the use of Photoshop technology for contrast improvement was signalled only once in 260 publications, suggesting that many authors ignored its existence.

(2) The second problem in the order of importance is small image size, usually combined with low magnification. All too often, virus images are penny- or stamp-sized. Unfortunately, journals are sometimes responsible for dwarf images as they may reduce image size to save space. No images should be smaller than 5 × 6 cm and tailed phages, such as lambda and T4, should have a particle length of at least 4 cm. Only so can image quality and the correct reproduction of details, such as tail striations, be assessed. In the case of isolated viruses, referees and journals should insist on final magnifications of at least 150,000×.

It is true that users of manual and digital electron microscopes face different challenges. On the one hand, the grain size of photographic films is smaller than the pixel size of most digital cameras and therefore users of most digital cameras must use higher initial magnifications than in photographic recording and subsequent enlargement, thus reducing fields of view. On the other hand, photographic enlargement is a pitiless procedure that exposes any flaws in the specimen and recording. However, these considerations are unimportant since it is only the result, a good or a poor micrograph, that counts in a publication.

**Figure 1:**
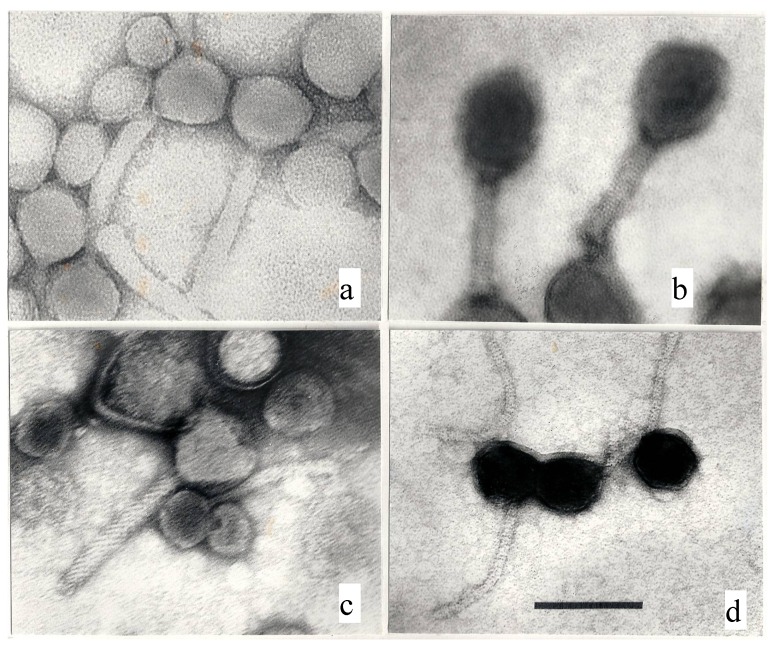
A rogue gallery of poor electron micrographs. (**a**) A contreastless image of a *Pseudomonas* myovirus; (**b**) Underfocused, unsharp image of two T4-like phages. (**c**) Underfocused, unsharp and astigmatic picture of a *Pseudomonas* myovirus, a siphovirus, and various cell debris; (**d**) Positive staining in a T5-like coliphage; note the apparent absence of edges and facets on the phage heads. 297,000×; uranyl acetate; the bar indicates 100 nm.

(3) Unsharp images may have many causes: dirty or misaligned lenses, objective lens astigmatism, specimen drift, over- or underfocussing, poor staining with stains of different thickness [13]. and dirty stain solutions or support film. These causes cannot be discussed here because they are generally not evident in the published phage images. Examples of problematic images are shown below (Figure 1a–c) or have been published elsewhere [14].

### 3.2. Human Responsibility

(1) The worst problem seems to be ignorance, namely that images of positively stained bacteriophages (Figure 1d) are mostly worthless and should be discarded. Positive staining, already observed in 1955 [2], is the staining of the virus particle itself, generally by uranyl acetate. Due to the affinity of UA to complexes of double-stranded DNA and protein [5], it produces high-contrast images of phage heads and is understandly popular with inexperienced investigators. Positive staining appears as a generally undesirable by-product of negative staining and cannot be reproduced at will. Its only use is in virus counts in water because positively stained viruses are easy to count at low magnification. The capsids of positively stained phage are always shrunken and structural details such as edges and capsomers are invisible [14]. Their dimensions are useless.

(2) Many manuscripts are incomplete with respect to materials and methods, not providing information on stains, magnification control, virus purification, measurements, and even (*horribile dictu*) the electron microscopes used. Others offer no virus dimensions and little or no comparison with similar and possibly related viruses in the literature.

(3) Misclassifications are frequent. They reflect ignorance and are totally avoidable. For example, in the literature and as a reviewer, I encountered papers or manuscripts where siphoviruses (characterized by noncontractile tails), were called “myoviruses” (characterized by contractile tails). I also saw micrographs of damaged viruses, with emtpy heads and/or broken tails, happily described as “new” phages. On many occasions, cell wall debris were described as “cystoviruses” or “tectiviruses” and bacterial flagella or pili were identified as “inoviruses”.

(4) Magnification control or calibration is described or mentioned in only 10% of publications. Its absence usually signals a poor-quality paper. Magnification is first adjusted at the installation of an electron microscope, to our reckoning mostly or always by means of beef liver catalase crystals [15]. Magnification does not remain stable over the whole lifetime of an EM (which may attain a proud 45 years as in the case of the author’s Philips EM300) and must be periodically checked and adjusted. Some authors seem to rely on scale markers printed on micrographs by some digital cameras. These scale markers, if uncontrolled, have no value whatsoever. Others investigators use diffraction grating replicas. Unfortunately, the latter are for low magnification (up to about 40,000×) and should be replaced by catalase crystals to obtain more reliable dimensions. Interestingly, especially for phage investigators, catalase crystals could be replaced by T4 phage tails, which are much easier to use [14].

(5) Viruses are often not purified in any way and image of crude lysates are offered instead, although excellent purification methods exist since a long time (e.g., CsCl density gradient centrifugation). As a consequence, preparations may be full of proteins and bacterial and phage debris. Clearly, sample preparation is a key for phage electron microscopy.

(6) Reviewers may be incompetent or just lazy. As a reviewer, I often noticed that referees were supremely knowledgeable in genomics and sequencing, but utterly incompetent in electron microscopy and unable to spot the most glaring faults. This is certainly a major reason why the phage literature now abounds in poor EM. See details in Table 4.

**Table 4:** Some reasons for poor quality.

Technical	Investigator-related	Journal
Image too small	Insufficient information on methods	Image too small
Magnification too low	Positive staining	Incompetent referees
Magnification not checked	Dirty samples	-
No contrast	Incomplete virus descriptions	-
Unsharp	No comparisons	-
No details visible	Misclassification	-

## 4. Reasons for Declining Quality

Is there a decline at all? Certainly as many, many present-day phage images are vastly inferior to the very first phage pictures, for example of coliphage T2, published in 1959 by Brenner *et al.* [3,4]. Poor and deteriorating electron microscopy is as pervasive as climate change. We have seen that poor EM is not linked to any country (with one possible exception), journal or electron microscope. Non-availability of scientific literature, formerly a serious problem in East European countries, is not a reason anymore since the advent of the Net. Clearly, poor EM is linked to the abilities of individual laboratories and caused by a multitude of factors, whether instrumental or dependent on the personal know how of individual electron microscopists.

How did the decline occur? (1) Part of the problem must be the disappearance of great electron microcopists who were role models and set standards, namely D.E. Bradley in Canada, E. Kellenberger in Switzerland, and A.S. Tikhonenko in Russia. (2) Other factors are an influx of unskilled operators, visible in the great number of new names in the field, and over-emphasis on genomics to the detriment of electron microscopy. For example, I heard the comment “It is not necessary to make good electron microscopy as long as there is a good sequence”. (3) Another possible reason may be a change in work habits. It appears in discussions that “manual” electron microscopists were careful to select images for good staining, focussing, purity of the sample, and astigmatism correction. I used myself to “hunt” for morphological aberrations and pentagonal capsids to prove icosahedral capsid symmetry. This attitude seems to have been lost in “digital” electron microscopists and the prevailing thinking seems to be that “shooting” 10–30 images will reveal all features of a phage. (4) Another possible reason for loss of quality, that foreshadows future decline, is the present explosive proliferation of periodicals, in particular of open-access journals. This has already led to perfunctory reviews and absence of quality control by publishers [16] and is certainly not a good omen.

What must be done? The key to preventing a general meltdown of quality, and indeed the only effective barrier against it, is the Referee-Editor system because investigators have to implement the reviewer’s recommendations. However, it depends essentially on the competence of the reviewers and their support by editors. Novel virus (phage) micrographs should be compared with existing virus atlases and high-quality publications at submission to a journal. The aim is a return to the *status quo ante*, that is the excellent quality of phage micrographs attained in the years 1970–1980. Otherwise, standards will continue to decline and investigators, referees, and journals will become more and more permissive. The logical end is a vicious circle.

## 5. Conclusions

What can be done? Remedies are numerous but not always applicable. Only a few of general nature can be discussed. Courses in electron microscopy will have little impact because of the great diversity of instruments in use, the small number (5–9) of attendants, and the absence of comprehensive manuals. Setting up a network of reference laboratories is likewise impractical. I tried precisely this and got nowhere. The main reasons were legal problems, fees, and modes of payment. By contrast, having an atlas of bacteriophages would be extremely helpful in phage identification and setting photographical standards. Indeed, the only phage atlases available are that of Dalton and Haguenau from 1973, mostly devoted to mammalian viruses [6], the book of Tikhonenko from 1968 [9], and an outdated, debatable collection of micrographs of enterobacterial phages [17]. See details in Table 5.

**Table 5:** Possible correctives.

Countermeasures	Effectiveness	Comments
Courses in EM	Little	Too many different instruments in use
Reference laboratories	Impractical	Legal and monetary problems
Atlas of bacteriophages	Desirable	Copyright problems
Standards for pictures	Easy	Must be adopted by journals
Contrast improvement	Easy or not	Manual EMs: use graded filters; Digital EMs: use Photoshop or the like
More severe reviews	Great	Must be required by journals
Signed reviews	Great?	Probably unpopular
Blacklisting poor referees	Dubious	An internal matter for individual journals

Some standards for pictures are simple enough. Pictures should be underfocussed and show a grainy background. Both PT and UA are equally good stains, but investigators are encouraged to use both stains for comparison. Resolution should be tested by means of T4 phage tails. Micrographs should reveal tail striations and fibers or spikes. T4 tail fibers have a diameter of slightly over 2 nm and T4 tail striations are about 4 nm wide. In all phages, micrographs should reveal tail striations and be sufficiently large in size [see Section 3.1., (2)].

Authors have the right to specify the size of their pictures. In my experience, this usually works. Phages must be intact and negatively stained while full dimensions must be given. Positively stained phages should not be shown unless in particle counts in aquatic microbiology or to show that positive staining is possible. Contrast of digital images may be improved by Adobe Photoshop C23 technology (San José, CA, USA) or similar software, e.g., Paint.NET and GIMP (“GNU Image Manipulation Program”) [18,19,20]. It is also noteworthy that the Gatan UltraScan 1000XP camera (Gatan, Pleasanton, CA, USA) offers the possibility of contrast enhancement. Grey images with no contrast should be systematically rejected.

Journals will have to set up guidelines and implement them. This may be the most difficult aspect of improving the situation. In essence, it amounts to a tightening of standards and more critical reviews. Signed or “open” reviews might help, but probably will never be popular. It may suffice if journals invite referees to devote particular attention to electron microscopy.
